# How the cell cycle enforces senescence

**DOI:** 10.18632/aging.101316

**Published:** 2017-10-30

**Authors:** Helena Silva Cascales, Erik Müllers, Arne Lindqvist

**Affiliations:** Department of Cell and Molecular Biology, Karolinska Institutet, Stockholm, Sweden

**Keywords:** G2, senescence, Cdk1, Cdk2, p21

Maintenance of genome integrity is essential for a cell. Upon DNA damage cells activate a signalling cascade to block cell cycle progression and initiate DNA repair. The initial steps of the DNA damage response (DDR) are mediated by the actions of ATM and ATR that lead to the activation of multiple factors, including p53 and p21. Together, these factors halt cell cycle progression by inhibiting cell-cycle activities as Cyclin-Cdk complexes and Plk1.

At the same time that cell-cycle activities are blocked by a DDR, they also have functions after DNA damage, among others in directing DNA repair. This may seem a paradox. Now we have found that a low level of cell-cycle activities is sustained after DNA damage. Together with the DDR, sustained cell-cycle activities stimulate cell-cycle exit and senescence from G2 phase.

Although a DDR is initiated, DNA damaged-cells that have passed the G1/S border continue to progress through S and G2 phases accumulating cyclins and Plk1 to mid-G2 phase levels [1, 2]. At this decision point, Cyclins are actively translocated into the nucleus and targeted for degradation by APC/C^Cdh1^, marking an irreversible transition to exit the cell cycle and become senescent. Interestingly, cells deficient in p21 or p53 do not present this decision point. While these cells still activate a cell-cycle arrest, Cyclins can keep accu-mulating to levels higher than observed during unperturbed mitotic entry [2]. Similarly, cells deficient in p53 or p21 reinitiate accumulating high levels of Plk1 and Cyclin-Cdk activities once ATM cannot sustain global phosphorylation on chromatin, eventually resulting in mitosis with unrepaired DNA damage [3].

**Figure 1:**
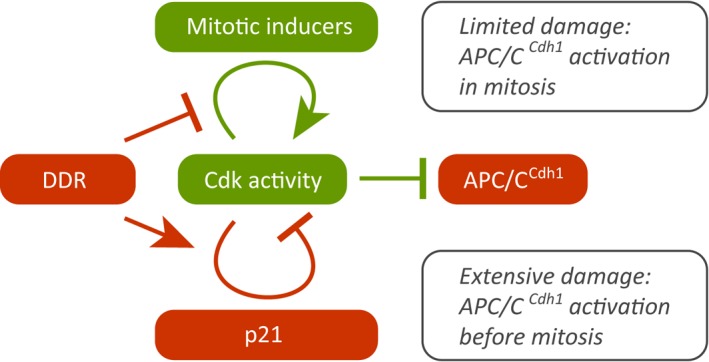
Cdk stimulates its own activity by several feedback loops, ultimately leading to mitotic entry. In the presence of DNA damage, Cdk also drives production of p21, which reduces Cdk activity and eventually leads to activation of APC/C-Cdh1. Active APC/C-Cdh1 degrades mitotic inducers and forces cell-cycle exit and senescence. Thus, after DNA damage, the signalling that if unchecked would drive mitotic entry ensures timely cell cycle exit and senescence.

Further investigating the mechanisms that drive induction of senescence in untransformed cells, we recently identified an apparent paradox in the DDR. In the presence of DNA damage, the DNA damage checkpoint represses Cdk activity to prevent mitotic entry of damaged cells. However, cells retain low levels of Cdk activity that support a slow progression through S and G2 phases allowing for DNA repair as well as expression of G2-specific proteins. In this situation, these low levels of Cdk activity are able to function as a decisive switch to determine cell fate. On the one hand, if cells are able to repair DNA damage efficiently, progression to mitosis is possible thanks to the slow yet steady accumulation of mitotic inducers primed by Cdk activity. On the other hand, if the damage is too exten-sive, we find that Cdk activity is able to induce p21 expression, which leads to cell cycle exit and onset of senescence from G2 phase [4] (Figure 1).

How p21 induces activation of APC/C^Cdh1^ remains unclear, but one alternative is that Cdk-mediated p21 expression constitutes a negative feedback loop that decreases Cdk activity to very low levels. In this case, Cdk-mediated inhibition of APC/C^Cdh1^ may not be sustained. Further, if Cdk activity is absent, the transcription of G2 factors is likely inefficient, leading to the loss of a biochemical G2 state over time. Coupling of Cdk activity to p21 expression ensures stronger induction of p21 if damage occurs in late G2 phase, where Cdk activity is highest. Thus inducing senescence and avoiding mitotic entry in the presence of DNA damage. In line, Feringa and colleagues recently found that cells in late G2 induce cell cycle exit earlier and at lower levels of DNA damage than cells in early G2 [5]. This infers that the time a cell has available to repair DNA lesions and reverse a checkpoint depends on its cell cycle position when the damage occurred, where cells in late G2 get little or no time at all. That Cdk activity is able to promote induction of senescence to prevent mitotic entry of damaged cells highlights the decisive role of Cdk activity for cell fate determination [4].

During an unperturbed mitosis, raising Cdk activities eventually lead to APC/C-dependent reset of the cell cycle. In this sense, cell-cycle exit in G2 phase is similar to a mitotic oscillation. However, as the Cdk activities are much lower after DNA damage, the oscillation of Cdk and APC/C does not lead to cell division. Recently, a similar mitotic oscillation with too low Cdk activity to lead to cell division was identified to drive the production of motile cilia during differentiation of brain cells [6]. This opens up for the possibility that low-level oscillations of mitotic factors regulate other events than ciliogenesis and cell cycle exit.

Even in the case of low levels of DNA damage that do not lead to cell cycle exit in G2, p21 expression is stimulated during G2 phase. After cell division, the retained p21 determines the levels of Cdk activity of the daughter cells, translating into different lengths of G1 phase. Thus, cells with sustained DNA damage will not be able to accumulate high levels of Cdk activity and will remain in a quiescent state after cell division [7, 8].

In conclusion, Cdk-mediated p21 production, leading to cell cycle exit in G2 or quiescence after cell division may act as a barrier to prevent genome instability and accumulation of mutations.
